# The rise of animal biotelemetry and genetics research data integration

**DOI:** 10.1002/ece3.9885

**Published:** 2023-03-16

**Authors:** Mara F. Müller, Sam C. Banks, Tara L. Crewe, Hamish A. Campbell

**Affiliations:** ^1^ Research Institute for the Environment and Livelihoods Faculty of Science and Technology, Charles Darwin University Northern Territory Darwin Australia; ^2^ Department of Natural Resources and Renewables Government of Nova Scotia Kentville Nova Scotia Canada

**Keywords:** animal tracking, biotelemetry, genetics, genomics, individual‐based movement, movement ecology

## Abstract

The advancement and availability of innovative animal biotelemetry and genomic technologies are improving our understanding of how the movements of individuals influence gene flow within and between populations and ultimately drive evolutionary and ecological processes. There is a growing body of work that is integrating what were once disparate fields of biology, and here, we reviewed the published literature up until January 2023 (139 papers) to better understand the drivers of this research and how it is improving our knowledge of animal biology. The review showed that the predominant drivers for this research were as follows: (1) understanding how individual‐based movements affect animal populations, (2) analyzing the relationship between genetic relatedness and social structuring, and (3) studying how the landscape affects the flow of genes, and how this is impacted by environmental change. However, there was a divergence between taxa as to the most prevalent research aim and the methodologies applied. We also found that after 2010 there was an increase in studies that integrated the two data types using innovative statistical techniques instead of analyzing the data independently using traditional statistics from the respective fields. This new approach greatly improved our understanding of the link between the individual, the population, and the environment and is being used to better conserve and manage species. We discuss the challenges and limitations, as well as the potential for growth and diversification of this research approach. The paper provides a guide for researchers who wish to consider applying these disparate disciplines and advance the field.

## Graphical abstract

The integration of movement and genetics data can broaden our understanding of the causes and consequences of individual‐based animal movement and provide insight into evolutionary and ecological processes. A review of the published literature found a recent trend toward integrating the analyses of the two different data collections, which has revealed novel insights. The review illustrates a rapidly growing research area and provides considerations and recommendations to guide researchers and advance the field.

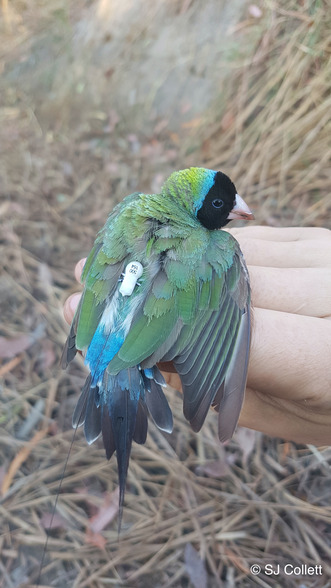

## 1 INTRODUCTION

Animal spatial movement is an integral component of ecological and evolutionary processes (Nathan et al., 2008). Elucidating the links between individual movement patterns and gene flow may assist in understanding how localized environmental conditions, animal behavior, and management strategies influence animal population trajectories and evolutionary processes (Fraser et al., 2015; Morales et al., 2010). As the actions of humans upon the natural world become ever more pervasive, there is an urgent need to improve our understanding of ecological processes by integrating information from multiple sources or fields of study as this will broaden our knowledge on the links between the different ecological levels (Zipkin et al., 2021). Technological advancement in animal biotelemetry devices now makes it possible to collect highly accurate and precise data on individual‐based movement at high frequencies and over long periods of time (Hussey et al., 2015; Kays et al., 2015). Analogously, the entire genome of a wild animal population can be sequenced, and the patterns of diversity, divergence, adaptation, and population history assessed rapidly and at low costs (Bourgeois & Warren, 2021; Lou et al., 2021). The relationship between the movements of individuals and the dispersal of genes has been recognized as key to understanding various ecological and evolutionary processes, and there is a growing body of literature that has integrated these two types of data collection within a single study (Cayuela et al., 2018).

In animal biotelemetry research, an animal's geographical location in its environment at a particular point in time is determined and logged using a variety of means (satellite‐based, node‐based, geolocation) without observer presence. The devices also carry other sensors so that complementary data (behavioral, physiological, and environmental) can be collected at similar spatiotemporal resolution (Taylor et al., 2017). These rich data collections are being used to improve the management of animal populations, through illustrating an individual's habitat requirements, space use, and associations (Brooks et al., 2019; de La Cruz et al., 2014; Suraci et al., 2020). However, the information provided by animal biotelemetry is individual‐based and thus limited by the temporal scale of the individual, making it challenging to understand the evolutionary or population‐level consequences of the observed movements (Hebblewhite & Haydon, 2010).

Genetics is linked to dispersal in several ways. Genetic variation within individuals, including variation at specific genes, has been associated with dispersal propensity or behavior (Shafer et al., 2011). Dispersal patterns are a key driver of the distribution of genetic variation within and among populations (genetic population structure; Shanahan et al., 2011). Consequently, genetic information at the level of populations or individuals (such as pairwise relatedness between individuals) can be used to understand kin interactions, connectivity between populations, and the impacts of individual movement events upon genetic diversity and population viability (Lemopoulos et al., 2019; Paetkau et al., 2004; Pierson et al., 2013). Therefore, genetic data can assist in quantifying dispersal patterns and understanding the consequences of individual‐based movement. Therefore, there are opportunities for expanding existing ecological theories through the integration of biotelemetry and genetics methodologies.

The merger of these fields is assisting us to better understand the genetic basis of adaptive behaviors like hibernation and elucidate fundamental ecological and evolutionary processes such as population responses to environmental change (Shafer et al., 2016). Here, we systematically reviewed the scientific literature to assess the breadth of research that has integrated the methods of biotelemetry with genetic information and the advances made from such integration. For each study, we determined the rational for the integration, the approach used, and the outcome that resulted from the combination of both data types. We report upon common trends, drivers of the research, and future opportunities that may assist in linking these largely disparate research fields and guiding future researchers.

## 2 METHODS

### 2.1 Systematic literature compilation and categorization

We carried out a literature search through January 2023. We used Web of Science, Scopus, and Google Scholar to discover published manuscripts that reported the use of both animal telemetry techniques and genetics methods. We carried out one search for each taxon (i.e., amphibian, bird or avian, fish, mammal, and reptile) using specific search terms for both telemetry and genetics (see Appendix 1). Since not all papers are openly available in a full‐text format, we only searched in the title, keywords, and abstracts. This returned a total of 398 manuscripts.

**FIGURE A1:**
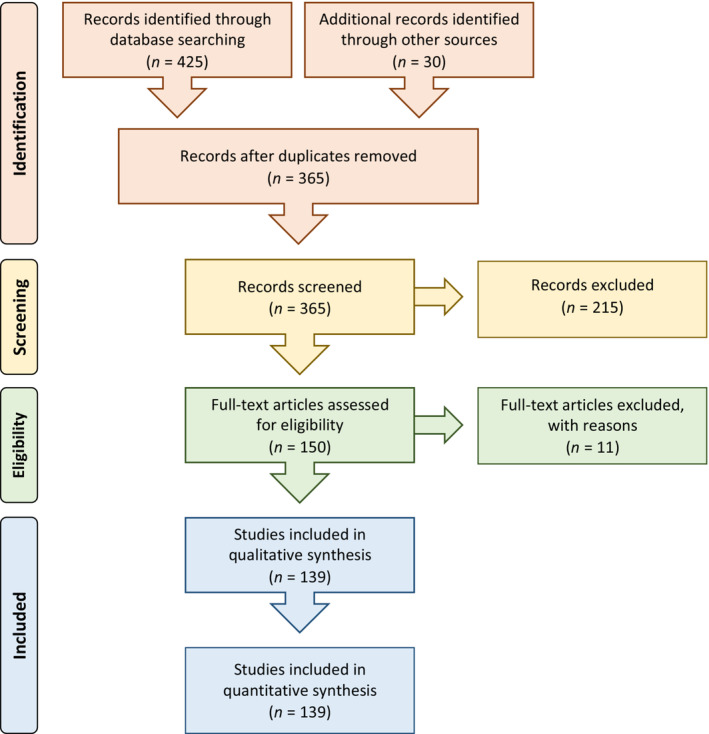
PRISMA diagram showing the number of papers included and excluded at each stage.

To further refine the Web of Science and Scopus search, we used the “revtools” package in R‐project (v 4.0.2; Westgate, 2019). We imported the 398 references into R studio (v 1.3.1056), removed duplicates, and used the *screen_abstracts* function to manually select only those papers where the title or abstract explicitly mentioned the use of both biotelemetry and genetics techniques. After the main author read the full text of the selected manuscripts, we kept 139 articles to be included in the analysis (Appendix 2: Figure A1).

To understand the scope of integrating biotelemetry with genetics, and whether the approach differs among taxa, we manually categorized these remaining articles into the following variables: aim of the study, duration of the study, sample size for telemetry and genetics, biotelemetry technology used, genetic markers used, genetic and biotelemetry statistics, global outcome of the study, and whether the study had a population‐level or individual‐level approach. Other information that we included was the publication year, the country of publication, and information on the study species (order, family, and environment). A previous perspective paper was used as a guide for determining the overall aim of the study derived from the integration of telemetry and genetics (Shafer et al., 2016). More detailed information on the categorization and classification criteria for some of the most relevant variables can be found in Appendix 3. To reduce the risk of bias, the main author carried out the initial categorization of all the articles and the secondary authors selected a random subset of the data and double‐checked the resulting classifications.

### 2.2 Quantitative data analysis

To carry out the quantitative data analysis, we used R‐project through the R studio interface (v 1.3.1056; R Core Team, 2021). We ran a discriminant correspondence analysis (DiCA) using the “TExPosition” package in R (Beaton et al., 2019) to analyze whether the variables of interest could be used to predict the taxon of the study species, thus indicating differences in the research approach. DiCA is an extension of discriminant analysis (DA) and correspondence analysis (CA) that categorizes observations in predefined groups (i.e., DA) using nominal variables (i.e., CA). Each group is represented by the sum of observations, and a CA is performed on the groups using a contingency table. The original observations are then assigned to the closest group, providing comparisons between the a priori and a posteriori classification to assess the quality of the model (Abdi, 2007). Since the DiCA function needs all variables to be nominal, we binned the temporal extent of the studies (four categories: 0, 2, 5, 10, 20 years), the sample size for genetics (seven categories: 0, 20, 50, 100, 200, 500, 1000, 3000ind) and for telemetry (seven categories: 0, 10, 20, 50, 100, 200, 500, 3000ind), and the year of publication (six categories: 1995, 2000, 2005, 2010, 2015, 2020, 2023). We then used all categorical variables of interest as the nominal variables and the taxon as the grouping variable, including only the three main taxa (birds, fish, and mammals) because the sample size was not sufficiently large for amphibians and reptiles.

After the DiCA confirmed notable differences in the research approach among taxa, we calculated frequencies for the categories of the most important variables using the *count* function within the “dplyr” package in R, and we inspected the temporal and spatial distribution of the studies.

## 3 RESULTS

**FIGURE 1:**
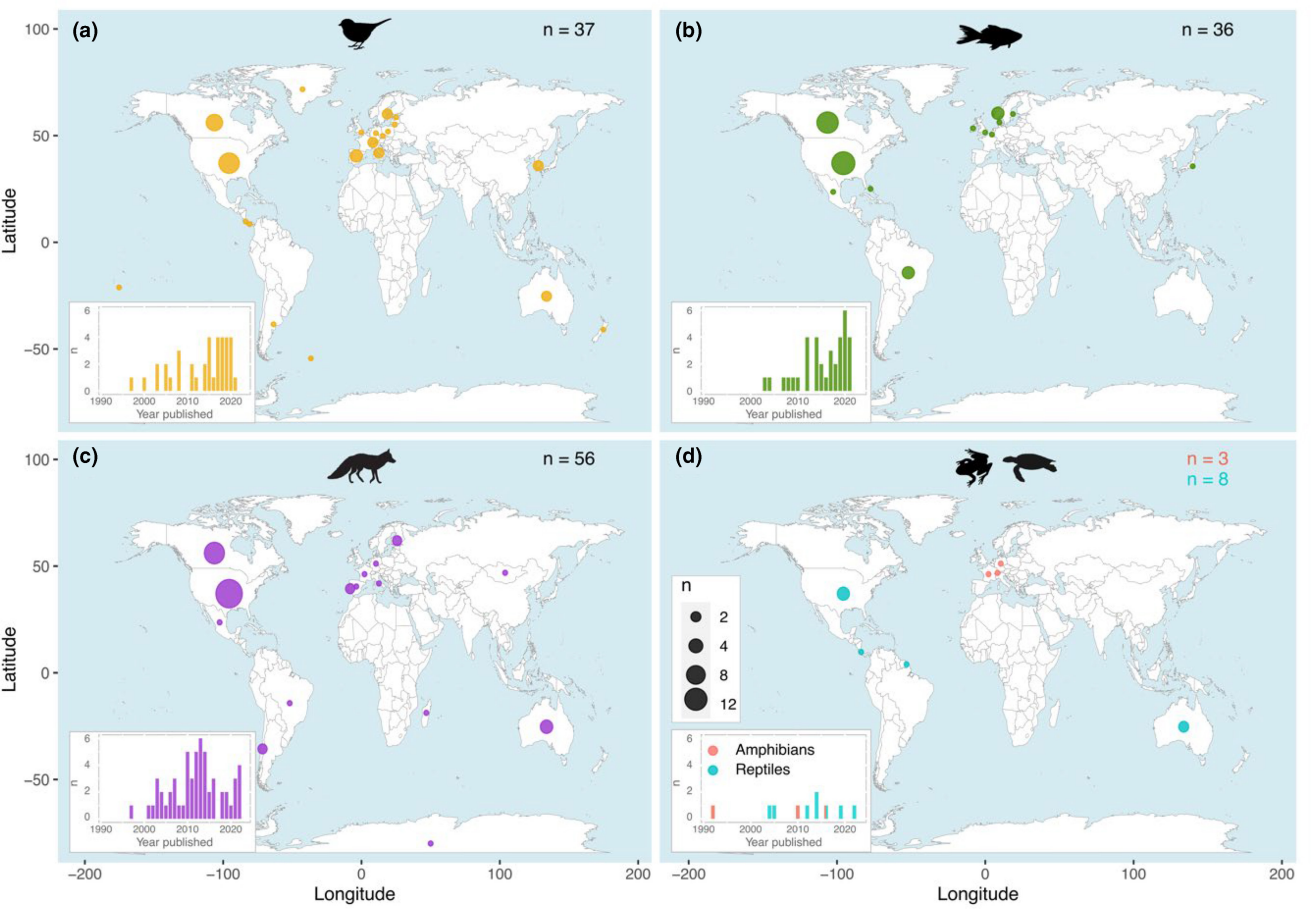
Distribution of bird (a), fish (b), mammal (c), amphibian, and reptile (d) studies according to the country where they were carried out and the year of publication. The overall sample size was 139, but one study had two study species belonging to different taxa (mammals and birds), thus adding up to 140.

**FIGURE 2:**
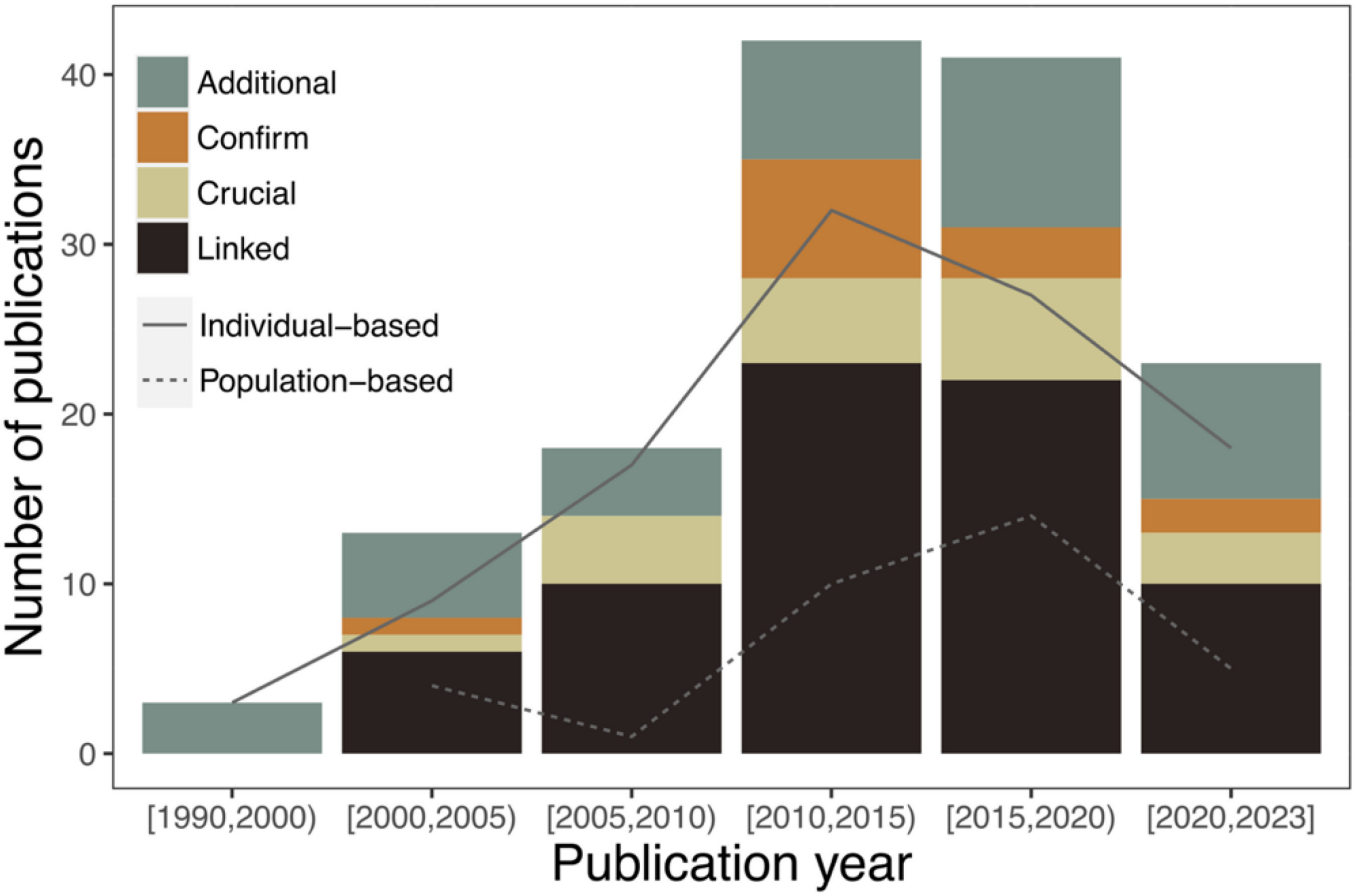
Number of publications within five‐year intervals for each study outcome category along with the linear trend of individual‐based and population‐based studies. Detailed descriptions of each category can be found in Figure 5.

The review returned 139 independent studies that had combined individual‐based movement data collected by biotelemetry with genetic information (Figure 1). Seventy‐five percent of the studies were published after 2010. The aim of the data combination for most of the studies was related to understanding animal movement, studying altruism and kin selection, and analyzing the flow of genes across the landscape as well as the impact of environmental change. Other research approaches, such as analyzing the link between movement patterns and gene expression or characterizing interspecific interactions, occurred at much reduced frequencies in the literature (Table 1). While all the papers included in this review collected genetics and individual‐based movement data, only 51% combined the two data collections in the statistical analyses (i.e., linked analyses). The remaining studies analyzed the genetics and telemetry data independently and then made inferences based upon the two separate outputs (i.e., nonlinked analyses). The integration of the two data collections showed a notable increase between 2010 and 2020 (Figure 2).

**TABLE 1:** Proportion of publications classified into each category according to the research aim that was addressed, the genetic markers used in the study, the statistical analyses used on the genetic data, the tracking technology, and the statistical analyses used on the telemetry data.

Taxon	Birds (*n* = 37)	Fish (*n* = 36)	Mammals (*n* = 56)	Total (*n* = 139)
Category name	Proportion of studies (%)			
Research aim				
Characterizing species interactions	5.4	0.0	5.3	2.9
Quantifying the impact of environmental change	5.4	27.8	12.3	15.1
Understanding animal movement	8.1	38.9	15.8	20.1
Gene flow and adaptive divergence	13.5	16.7	12.3	15.1
Altruism and kin selection	13.5	0.0	35.1	18.7
Mechanisms of pathogen transmission	8.1	5.6	7.0	6.5
Genotype: phenotype correlations	19.0	5.6	5.3	9.4
Gene expression	2.7	2.8	0.0	1.4
Mating systems as drivers of movement	24.3	2.8	7.0	10.8
Genetic markers				
DNA fingerprinting (minisatellites)	5.4	0	3.5	2.9
Microsatellites (simple sequence repeats)	59.5	52.8	78.9	65.5
Mitochondrial DNA (mtDNA)	10.8	16. 7	10.5	13.7
Single‐nucleotide polymorphisms (SNPs)	5.4	19.4	3.5	8.6
Pathogen genes	8.1	5. 6	0	3.6
Target genes	16.2	11.1	1.7	7.9
Other	2.7	2.8	7.0	5.7
Genetic statistics				
Population‐level genetic structure	16.2	44.4	19.3	27.3
Individual‐level spatial genetic structure	10.8	8.3	45.6	25.2
Genetic tagging	0	2.8	3.5	2.2
Individual assignment to populations	62.2	55.6	47.4	54.0
Genetic covariates of dispersal data	21.6	2.8	3.5	8.6
Taxonomic classification	10.8	11.1	3.5	7.2
Gene expression	2.7	5.6	0	2.2
Pathogen screen	8.1	5.6	1.7	4.3
Tracking technology				
Acoustic	0.0	55.6	1.7	12.4
Geolocator	18.9	0.0	0.0	5.8
PIT‐tag (passive integrated transponders)	0.0	13.9	0.0	3.3
Proximity loggers	0.0	0.0	10.5	5.0
Satellite	29.7	0.0	19.3	19.0
VHF	54.0	50.0	77.2	64.4
Telemetry statistics				
Home range	27.0	33.3	63.1	43.9
Dispersal	45.9	61.1	21.0	40.3
Behavioral activity	29.7	13.9	26.3	24.5

*Note*: Proportions were calculated for each of the three main taxa (birds, fish, and mammals) and for the overall (including amphibian and reptile studies). Studies could fall into more than one category for some variables, thus not adding up to 100%. The category “Other” within genetic markers includes markers that were present in <2 studies (whole genome, polymorphic loci, transcriptomics, and allozymes).

Most studies were carried out in North America and Europe, and the most abundant taxon was the mammalian. Sixty percent of all studies were carried out in North America, 25% in Europe, 6% in South America, 6% in Oceania, and 4% in Central America. Only one study was based in Africa and four in Asia, together representing less than 5% of all studies (Figure 1). Out of the 139 publications, 40% focused on mammals, 27% on birds, 26% on fish, 6% on reptiles, and 2% on amphibians. The total body of research covered between 7 and 12 orders across the three main taxa, although the distribution between orders was strongly biased. Forty‐two percent of all fish research focused on freshwater and another 42% on anadromous species (mainly salmonids and sturgeons), 47% of mammal studies focused on the carnivore order, and 51% of bird studies on the passerine order (Table 2). Only three studies had a multiple‐species approach, and they all had two study species. Most mammal studies were published between 2010 and 2014, whereas we observed a notable uptake in fish and bird research between 2015 and 2020 (Figure 1).

**TABLE 2:** Classification of the publications according to the Order (uppercase) and Family (lowercase) of their study species for the three main taxa (birds, fish, and mammals).

Birds (*n* = 37)	*N*	Fish (*n* = 36)	*N*	Mammals (*n* = 56)	*N*
**ACCIPITRIFORMES**	**2**	**ACANTHURIFORMES**	**1**	**ARTIODACTYLS**	**13**
Accipitrids	2	Drums	1	Bovids	2
Anseriformes	**3**	**ACIPENSERIFORMES**	**6**	Camels	1
Ducks	3	Sturgeons	6	Deer	7
**CHARADRIIFORMES**	**2**	**CARCHARHINIFORMES**	**1**	Pigs	1
Sandpipers	1	Requiem sharks	1	Porpoises	1
Skuas	1	**CHARACIFORMES**	**2**	Whales	1
CUCULIFORMES	**3**	Flannel‐mouthed characins	2	**CARNIVORES**	**27**
Cuckoos	3	**CYPRINIFORMES**	**2**	Bears	7
GALLIFORMES	**2**	Catfishes	1	Canids	2
Phasianids	2	Cyprinids	1	Cats	4
GRUIFORMES	**2**	**ESOCIFORMES**	**1**	Mephitids	1
Cranes	2	Esocids	1	Mustelids	7
PASSERIFORMES	**19**	**GADIFORMES**	**2**	Procyonids	3
American sparrows	1	Cods	2	Seals	3
Australasian wrens	1	**LABRIFORMES**	**1**	**CHIROPTERS**	**3**
Crows	3	Parrotfishes	1	Bats	3
Icterids	1	**PERCIFORMES**	**2**	**DIDELPHIMORPHS**	**3**
Manakins	1	Percids	1	Opossum	3
New world warblers	4	Serranids	1	**PERISSODACTYLS**	**1**
Silky flycatchers	1	**PETROMYZONTIFORMES**	**1**	Horses	1
Swallows	2	Northern lampreys	1	**PRIMATES**	**1**
Thamnophilids	1	**SALMONIFORMES**	**16**	Lemurs	1
Thrushes	3	Salmonids	16	**RODENTS**	**9**
Tits	1	**SILURIFORMES**	**1**	Cavies	1
Tyrant flycatchers	1	Long‐whiskered catfishes	1	Hares	1
PETRELS	**1**			Heteromyids	2
Procellariids	1			Octodontids	2
PSITTACIFORMES	**1**			Squirrels	3
Cockatoos	1				
STRIGIFORMES	**1**				
True owls	1				

*Note*: Some publications in the mammalian taxon included two study species, thus not adding up to the total number of publications. Bold text corresponds to the name of the variable, and underneath are the names of the categories within that variable.

**FIGURE 3:**
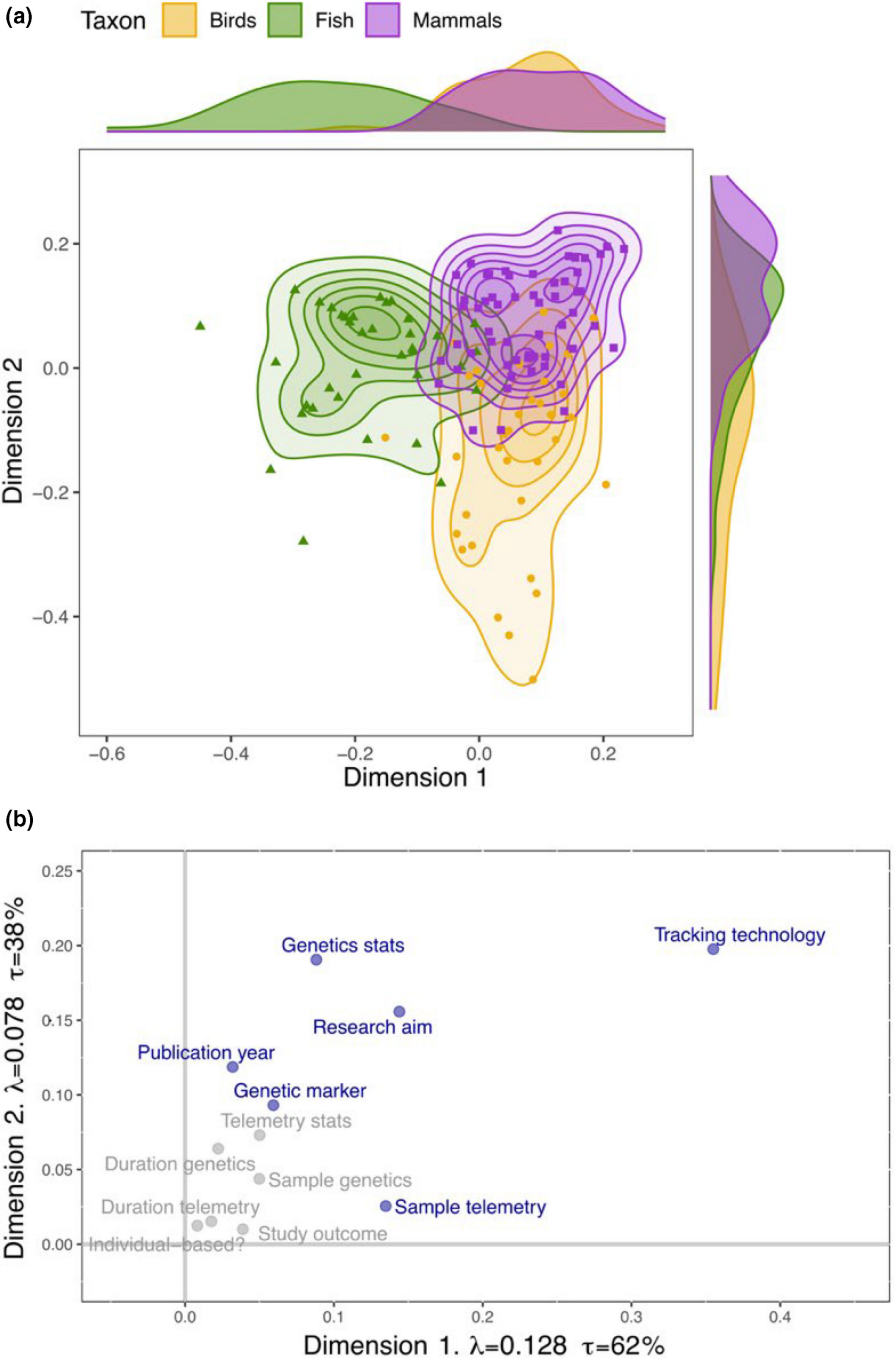
Discriminant correspondence analysis (DiCA) outcomes. (a) Distribution of latent variable data points inferred from observed data, grouped according to taxa, along with marginal density plots. (b) Contribution of each variable included in the DiCA model to the two dimensions of the data. Those variables explaining a significant amount of the variance in both dimensions are colored in dark blue. The fixed model, used as the training set, showed a classification accuracy of 84%, whereas the random model, used as the validation model (leave‐one‐out), had an accuracy of 70%. *λ* = eigenvalues, *τ* = variance explained (%).

DiCA results illustrated significant differences in the research approach among taxa (Figure 3). We found the greatest overlap between birds and mammals, which caused a considerable number of misclassifications in the random model (9/36 for birds and 14/57 for mammals). The main differentiating variable was the tracking technology used, followed by the research aim and the genetics statistics, and, to a lesser extent, the publication year, the genetic marker used and the sample size for the telemetry data (Figure 3).

The differences in research interests among taxa were linked to the use of different methodologies (Table 1). In birds, data were mainly used to better understand mating behaviors or to analyze the correlations between phenotypes and genotypes. For this purpose, genetic data were predominantly used to carry out assignment tests and to analyze covariates of dispersal and telemetry data were collected for dispersal measures, followed by activity pattern analyses. Fish research primarily focused upon understanding their movement and quantifying the impact of environmental change on their populations. Therefore, many researchers analyzed population‐level genetic structuring to understand population dynamics or assigned individuals to putative populations as an indirect dispersal measure. Telemetry data were predominantly used as direct measures of dispersal to contrast genetic results. A significant proportion of mammalian studies investigated altruism and kin selection. This necessitated the use of telemetry data for home‐range analyses, which were complemented with relatedness measures using assignment tests. To a lesser extent, mammalian studies also focused on the spatial distribution of individuals and genes to understand their movement, quantify the impact of environmental change and analyze their gene flow. Therefore, population‐level and individual‐level genetic structure analyses were also common, complemented by direct measures of dispersal through tracking data (Table 1).

**FIGURE 4:**
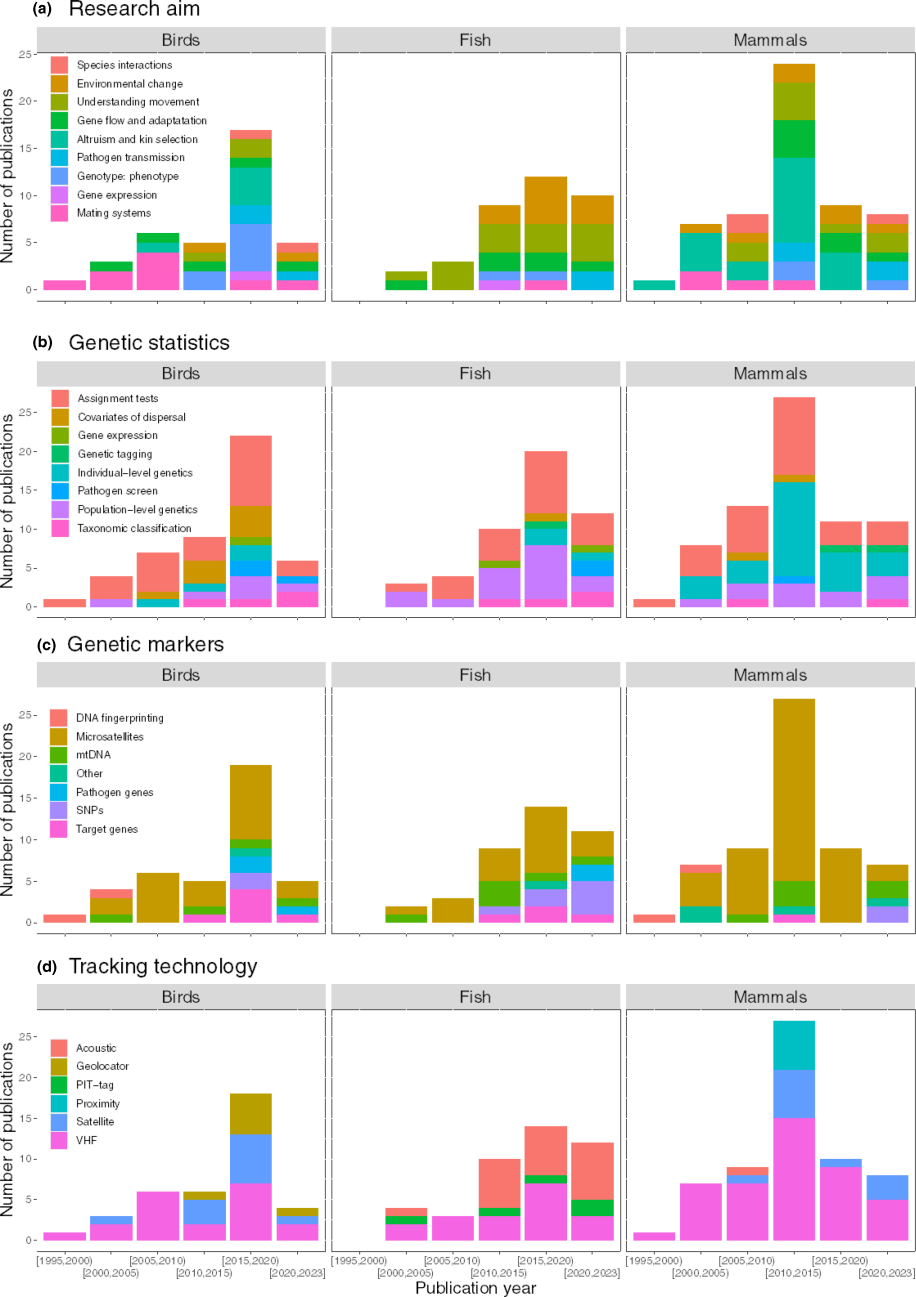
Temporal distribution of publications according to the research aim (a), the statistical analyses used on the genetic data (b), the genetic markers (c), and the tracking technology used (d) for the three main vertebrate taxa (birds, fish, and mammals). For some variables, the studies could fall into more than one category, thus not adding up to the total number of papers for each taxon. The category ‘Other’ within Genetic markers includes markers that were present in <2 studies (whole genome, polymorphic loci, transcriptomics, and allozymes). The statistical analyses used on the telemetry data did not show any clear temporal patterns, thus not being included in the figure.

The research interests and the methodologies that were used also changed over the years (Figure 4). Sixty‐four percent of the researchers used radio (VHF) tracking devices to collect the animal movement data, but since 2010 passive acoustic telemetry has increased in use for monitoring fish, geolocator tracking for birds and satellite telemetry has been more widely used for bird and mammal studies. The most used genetic markers were microsatellites (65%), followed by mtDNA (14%), but other markers such as single‐nucleotide polymorphisms (SNPs) or specific target genes were being cited more frequently in recent years. Results also showed a clear increase in individual‐level approaches, coupled to the increase in linked data analyses (Figure 2). Detailed illustrations on the temporal trends of the research aims and methodologies can be found in Figure 4.

**FIGURE A2:**
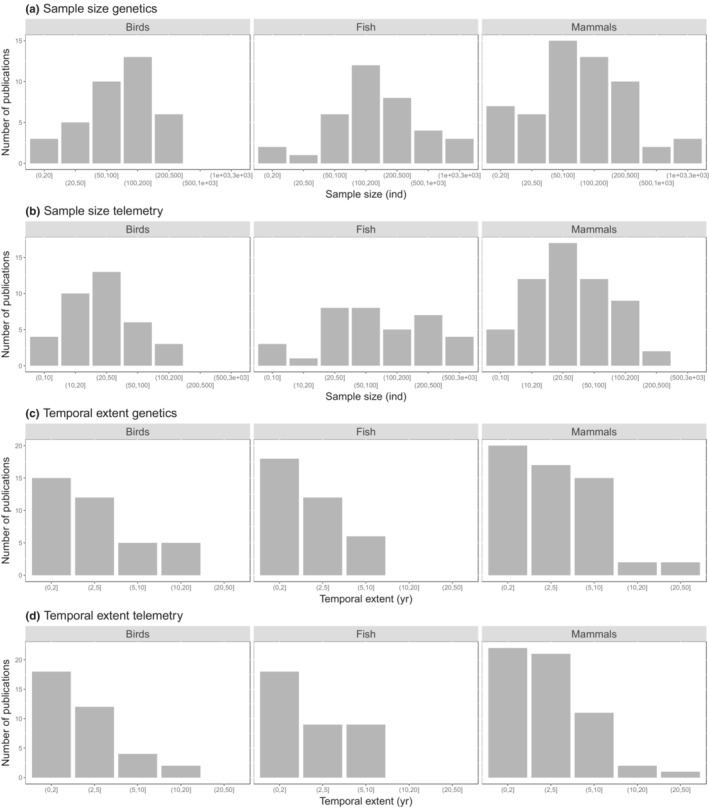
Distribution of studies according to the sample size for the genetics (a) and telemetry (b) data, and the temporal extent for the genetics (c) and telemetry (d) data collection.

The temporal extent of the data collection for most studies oscillated between 0 and 10 years both for the genetic data (92% of studies) and the telemetry data (96% of studies). Forty‐two percent of studies had a genetic sampling period of 0–2 years, whereas 47% collected telemetry data during 0–2 years. However, the average sample size was considerably bigger for the genetic data (most common categories: 28% 100–200ind, 22% 50–100ind, and 20% 200–500ind) compared with the telemetry data (most common categories: 30% 20–50ind, 20% 50–100ind, and 18% 10–20ind). Fish and mammal studies had genetics sample sizes of up to 3000ind, whereas the maximum sample size for birds was found to lie between 200 and 500ind. The maximum telemetry sample size was much bigger for fish (11% 500–3000ind, 19% 200–500ind, and 14% 100–200ind) compared with birds (0% 500–3000ind, 0% 200–500ind, and 8% 100–200ind) and mammals (0% 500–3000ind, 4% 200–500ind, and 16% 100–200ind; Appendix 4: Figure A2).

## 4 DISCUSSION

**FIGURE 5:**
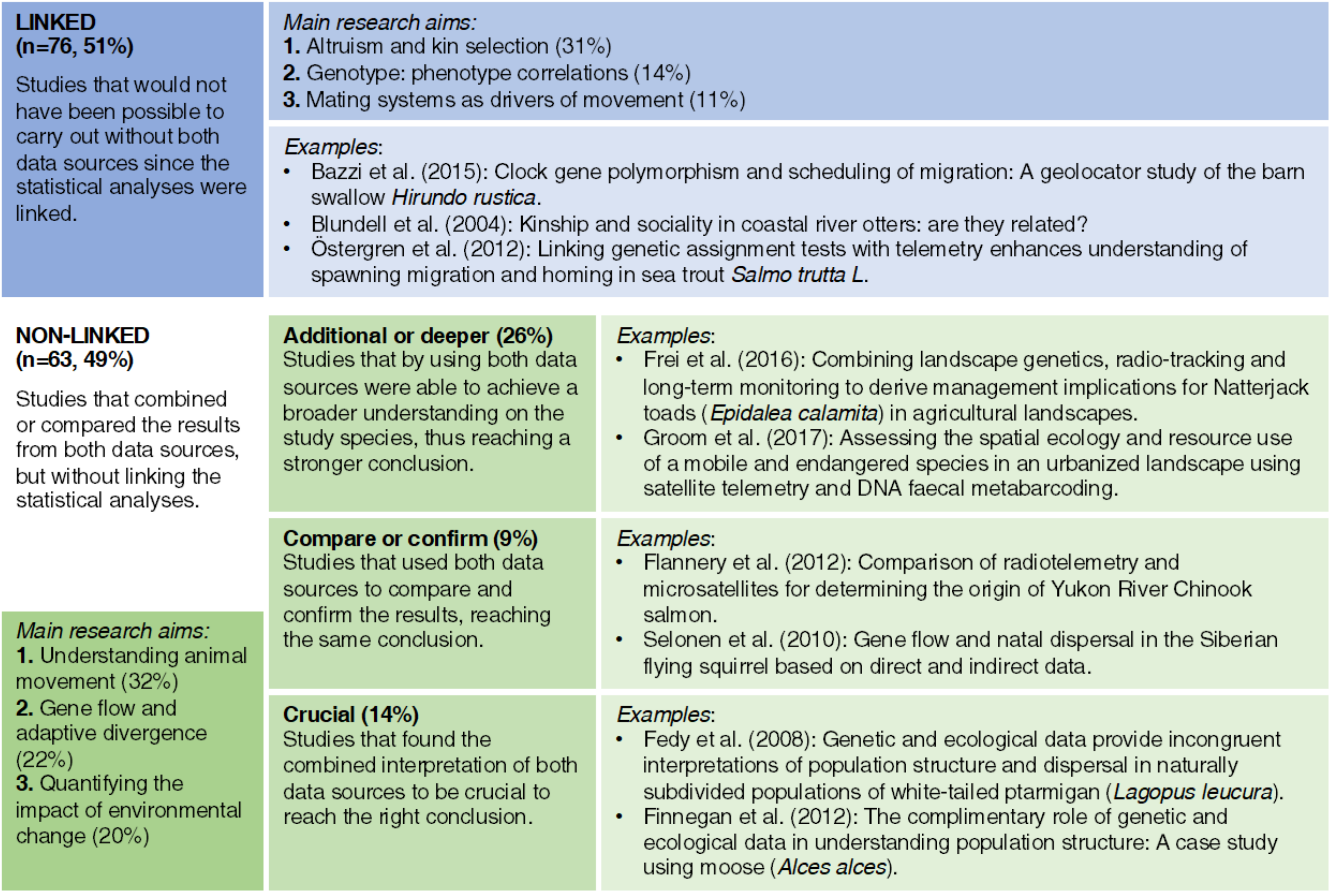
Proportion of studies falling within each outcome category along with descriptions and examples. The percentages for the overall outcome categories are for the total number of studies (*n* = 139), whereas the research aim percentages are within each category (linked/nonlinked).

The review found that the research combining telemetry and genetics data in their methodology is expanding and diversifying in its approach. There has been a paradigm change in how individual‐based movement and genetics data are being integrated and applied, suggesting that this emerging field has a dynamic and productive future. In about 50% of the studies, animal telemetry and genetic data were analyzed and interpreted using independent methods specific to each research discipline (i.e., nonlinked; Figure 5). This research used each dataset to validate, compare, or expand the findings from the other data source—generally around population connectivity (Fedy et al., 2008; Finnegan et al., 2012; Riley et al., 2006). Even if the statistical analyses were not directly linked, using both data types highly benefitted the outcome of the study and sometimes was crucial to draw the right conclusion. For example, animal telemetry data showed that up to a third of individuals from a subpopulation of bobcats (*Lynx rufus*) and coyotes (*Canis latrans*) regularly crossed over a large freeway, yet the populations on either side remained genetically differentiated (Riley et al., 2006). Thus, analyzing both data types enabled researchers to determine where effective dispersal was or was not occurring and the barriers to dispersal, which can be vital for assessing management strategies (Cayuela et al., 2018). This highlights how genetic and telemetry data generally inform upon processes happening at different temporal scales, hence why their combined interpretation can significantly broaden the knowledge gained on a population.

In manuscripts published after 2010, the two data types were increasingly combined within a single data plan and analysis structure (i.e., linked; Figures 2 and 5). This integrative methodology facilitated the use of individual‐based approaches to study the interrelation between fine‐scale genetic information and spatial behaviors. For example, using this approach it was possible to detect genetic and social structuring at fine spatial and temporal scales in white‐tailed deer (*Odocoileus virginianus*; Miller et al., 2010). To do so, the authors used spatial autocorrelation analyses to quantify the correlation between genotype frequencies and the pairwise geographic distance of individuals, averaging allele frequencies over loci for all pairs of individuals separated by geographic intervals of 200 m, as determined by the telemetry data. In another study, it was possible to establish a connection between genetic relatedness and mitochondrial haplogroups and migration timing in mule deer (*Odocoileus hemionus*), using regression models and Mantel tests (Northrup et al., 2014). Migration timing referred to the initiation and termination dates of migration events and was calculated from GPS tracking data. Similarly, tests of association were used to establish a connection between *Clock* gene polymorphism and migration traits in barn swallows (*Hirundo rustica*), derived from light‐level geolocator tracking (Bazzi et al., 2015). Following this line, many studies used genetic data for genotyping individuals and then studied the differences in the movement ecology between genotypes. This enabled researchers to detect differences in feeding and movement behaviors among coexisting ecotypes of Atlantic cod (*Gadus morhua*), using linear models (Kristensen et al., 2021). Moreover, several studies analyzed mechanisms of pathogen transmission, using genetics to detect infected individuals (screening for pathogen genes) and telemetry to track their movement and better understand pathogen spread (Gamble et al., 2020). An extension of the Mantel test called multiple regression quadratic assignment procedures (MRQAP) was used for more complex analyses, such as studying the effect of several factors, including genetic relatedness, on social structure (derived from telemetry data) in raccoons (*Procyon lotor*; Hirsch et al., 2013). Despite the greater mathematical complexity and difficulty in interpretation, we also found that the use of Bayesian statistics is on the rise (Converse et al., 2012; Finlay et al., 2020). Finally, the genetic‐telemetry data integration was also used to explain how environmental heterogeneity can affect population‐level processes in mountain goats (*Oreamnos americanus*; Shafer et al., 2012) and bobcats (*L. rufus*; Reding et al., 2013) based on how individuals interact with the landscape. These studies used the telemetry data to fit resistance surfaces and then tested the best predictor model for genetic relatedness using Mantel tests or multiple regression. The power of the genetic‐biotelemetry data integration to reveal the link between the individual, the population, and the environment makes this growing field extremely valuable in understanding and addressing some of the future's most challenging environmental problems (Grimm et al., 2017).

### 4.1 Taxon‐specific approach

The literature showed a significant divergence in the research themes among taxa. In mammals, the integration of biotelemetry and genetics was predominantly adopted to examine the relationship between kinship and spatial organization (Bartolommei et al., 2016). Such findings were often explained within a human‐centric focus (Kessler et al., 2014). Studies on birds largely focused upon extra‐pair paternity, widespread in birds (Brouwer & Griffith, 2019), establishing links between the movement behaviors of the parents and the parentage of their offspring (Stutchbury et al., 2005). However, there was also an increasing body of work focused upon understanding the genetic basis of migration by analyzing the link between genotypes (e.g., *Clock* gene regulation), and the timing, duration, or direction of migration (phenotypes; Bazzi et al., 2015). Integrating tracking data into studies on physiology and genetics is essential to foresee the consequences of environmental change on migratory bird species (Fudickar & Ketterson, 2018).

Fish research mainly studied the interconnection between movement patterns and stock structuring to make better informed management decisions around harvesting and fisheries exclusion zones (Dugo et al., 2004). There was, however, a growing body of research studying the impacts of in‐stream barriers upon individual movements and using genetic structure as an index of effective population size and health (Junge et al., 2014). This is in accordance with a boom in dam constructions, particularly in developing countries (Zarfl et al., 2015). Most reptile studies were upon marine turtles and mainly aimed at studying demography, population status, and habitat use (Troëng et al., 2005). All three amphibian studies were on the order Anura and studied the impact of environmental change on population connectivity (Safner et al., 2011).

We found that almost no publication had a multispecies approach. A previous review on wildlife connectivity research based in South Asia also highlighted the lack of studies including multiple study species (Thatte et al., 2021). The multispecies approach is significantly constrained by economic and technological limitations but can be crucial to implement effective landscape‐level conservation management strategies. However, the rapid evolvement of new technologies together with new approaches, such as species‐agnostic modeling (Marrec et al., 2020), should make it easier to address this research gap in the near future.

DiCA results clearly illustrated that the main differentiating variable between taxa was the biotelemetry technology. This could raise the question as to whether the research questions drive the methodological approaches or the other way around. It has previously been discussed how the rapid improvement of tracking technologies such as GPS technology can cause researchers to monitor wildlife simply because the technology is now available rather than to address specific biological questions (Hebblewhite & Haydon, 2010; Latham et al., 2015). This may lead researchers to prioritize research approaches that are most suited for the specific tracking technologies available for each species rather than focusing on achieving a mechanistic understanding on why animals behave in a certain way. Therefore, a research study should be carefully planned out and thought through before proceeding to collect any data (Latham et al., 2015).

### 4.2 Research trends

We found that the majority of studies had been published in high‐income countries. This is not unusual as animal biotelemetry and genetic sequencing technologies are reasonably expensive, making it challenging for researchers from low‐income countries to simultaneously adopt these two techniques (Kozakiewicz et al., 2018; Roberts et al., 2016). A mechanism to broaden the dual application of genetics and biotelemetry would be for researchers to improve public data archiving and sharing of each of the independent data collections. Sanger sequencing data, especially for mitochondrial DNA studies, have been shared through GenBank (https://www.ncbi.nlm.nih.gov/genbank/) for decades, providing a platform for collaboration and data access that has advanced the field of genetics. Data repositories for genetic marker studies (SNPs) are more challenging to share and compare since such datasets employ genetic markers that are typically species‐specific. However, whole‐genome sequencing techniques are on the rise in population genetics and the increased application of whole‐genome sequencing and the development of statistical analyses that can take advantage of such data for ecological and evolutionary questions will facilitate the growth of this field and the ability to synthesize across datasets. Similarly, there has been significant rise in the usage of shared repositories of animal biotelemetry data (Campbell et al., 2015; Taylor et al., 2017), as well as animal biotelemetry hardware and infrastructure (Crewe et al., 2020). This is being facilitated by a drive toward common data structure and formats (Campbell et al., 2016) and an increased desire to share data by younger researchers (Campbell et al., 2019).

In nearly all studies, the tissue or blood samples for genetic analysis were taken when the animal was captured for attachment of the telemetry device, thus requiring little extra effort and cost but benefitting the interpretation of the causes and consequences of the individual‐based movements (Donaldson et al., 2014). The increasing trajectory of publications that have integrated individual‐based movement and genetics data suggests this field is going to grow significantly over the next decade. For example, promising and powerful approaches such as the combination of nonlethal transcriptomics with individual tracking in fish have been emerging in recent years (Jeffries et al., 2021). This data integration is particularly relevant in actively managed populations, where using only observational data is generally not sufficient to infer population connectivity or detect dispersal events (Corlatti et al., 2009). A recent review on animal connectivity research found that most habitat suitability models using genetic data are lacking information on functional connectivity, highlighting the need for including data that informs how animals move through anthropogenic landscapes (Thatte et al., 2021).

### 4.3 Future directions

The observed taxonomic differences in the literature appeared to be driven not only by an application or need for the research findings but also by biotelemetry technology. The attachment of biotelemetry devices used to be constrained toward larger animals, but the size and weight of devices has significantly reduced in recent years to enable the tracking of ever smaller individuals. The advent of geolocator technology has now made it possible to even track the migratory movements of small‐bodied birds over long time periods (Fudickar et al., 2012). Similarly, tracking of marine animals that do not surface was not possible a few decades ago because radio waves cannot propagate in saltwater (Hussey et al., 2015). The advent of underwater passive acoustic telemetry has dramatically improved our ability to track the individual‐based movements of these animals. The continued reduction in the size of biotelemetry devices, their increased sophistication, and improvements in accuracy, precision, and longevity (Hussey et al., 2015; Kays et al., 2015) have significantly broadened the species that are studied using biotelemetry devices and enriched the data collected. The increased miniaturization of biotelemetry devices and sensors plus improvements in remote data transfer are enabling higher frequency and accuracy in the monitoring of a free‐ranging animal physiology and its environment. This is going to present significant opportunity for genetic data integration to help us better understand the interplay between an animal's phenotype, fitness, and plasticity to environmental change.

The widespread adoption of ddRAD and similar SNP “genotyping by sequencing” approaches in population genetics over the past decade have reduced the protocol development time for new species. Moreover, such techniques have become available through commercial providers to researchers without access to well‐equipped laboratories (Kilian et al., 2012), thus making them more affordable. This is resulting in a growing and exciting field with potential for cross‐fertilization between what were once disparate and unconnected fields of research. A clear example of the potential derived from these new technologies is the increase in fish population structure and migration research over the past decade, and its application to sustainable harvest (Jeffries et al., 2021; Thorstensen et al., 2022).

A perspective paper published in 2016 (Shafer et al., 2016) suggested that advancement in animal biotelemetry and genomics technology would significantly increase our understanding of animal ecology. This review demonstrates that this is indeed the case. We found that the four predominant research areas that were being addressed focused on understanding the spatial and temporal distribution of individuals and their genes, as well as on understanding social dynamics, and had been described by Shafer et al. (2016). However, some of the research areas previously described were hardly studied. The less prevalent approaches were characterizing species interactions and analyses of gene expression, which are probably most affected by the limitations in genetic data collection. Advancements made in genetic techniques such as high‐throughput environmental DNA (eDNA) and new sequencing technologies should increase data availability and provide new research opportunities.

## 5 CONCLUSION

The combination of telemetry and genetics data requires little extra sampling effort because a small tissue sample can be easily taken when the animal is captured for telemetry device attachment. The literature demonstrates that the ecological and evolutionary knowledge upon the study species, and its usefulness for conservation and management, is far greater when the two techniques are combined than when used in isolation. This review shows that the field is continuing to grow and provide information that will support better decisions around reversing the causes of animal population decline.

## AUTHOR CONTRIBUTIONS

**Mara F. Müller:** Conceptualization (equal); data curation (lead); formal analysis (lead); investigation (equal); methodology (lead); resources (equal); software (equal); validation (equal); visualization (lead); writing – original draft (lead); writing – review and editing (equal). **Sam Banks:** Conceptualization (equal); formal analysis (equal); investigation (supporting); methodology (supporting); resources (supporting); software (supporting); supervision (supporting); validation (equal); writing – review and editing (equal). **Tara L Crewe:** Conceptualization (equal); formal analysis (supporting); investigation (supporting); methodology (supporting); resources (supporting); software (supporting); validation (supporting); writing – review and editing (equal). **Hamish A Campbell:** Conceptualization (equal); formal analysis (supporting); funding acquisition (lead); investigation (supporting); methodology (supporting); project administration (lead); resources (supporting); supervision (equal); validation (equal); writing – original draft (supporting); writing – review and editing (lead).

## Data Availability

The data that support the findings of this study are openly available in “Dryad” at https://doi.org/10.5061/dryad.hx3ffbggs.

## APPENDIX 1 Literature search criteria.

The search terms used to select the papers combining biotelemetry and genetics data were as follows: “*telemetry” OR “Argos” OR “PTT” OR “*tracking” OR “VHF transmitter” OR “radio transmitter” OR “acoustic transmitter” OR “GPS animal track*” OR “GPS satellite track*” OR “movement ecology” OR “global location sensor” OR “geolocation” AND “genetic*” OR “DNA” OR “microsatellite” OR “single nucleotide polymorphism” OR “SNP” OR “molecular.” One search for each taxon was carried out, by adding AND Name of Taxon (i.e., amphibian, bird or avian, fish, mammal, and reptile). To get the broadest search, no quotation marks were used for the name of the taxon. “PTT” stands for platform transmitter terminal, “VHF” for very high frequency, and “SNP” means single‐nucleotide polymorphisms.

## APPENDIX 3 Informative table for variable categorization.

**Table:**

Category	Description
Research aim	
Characterizing species interactions	Studies aimed at analyzing interspecific interactions.
Quantifying the impact of environmental change	Studies aimed at analyzing how environmental change affects population processes.
Understanding animal movement	Studies aimed at studying how animals move to respond to their environment (e.g., dispersal or migration movements).
Gene flow and adaptive divergence	Studies aimed at understanding how the landscape affects the flow of genes.
Altruism and kin selection	Studies aimed at analyzing the relationship between genetic relatedness and social structure.
Mechanisms of pathogen transmission	Studies aimed at understanding host‐pathogen dynamics.
Genotype: phenotype correlations	Studies aimed at analyzing the correlation between phenotypic and genotypic variation.
Gene expression	Studies aimed at linking gene expression patterns to phenotypic differences.
Mating systems as drivers of movement	Studies aimed at understanding reproductive strategies through the study of individual movement patterns and measures of relatedness.
Telemetry statistics	
Home range	Analyses focusing on examining how animals are using the space.
Dispersal	Analyses focusing on distance measures.
Behavioral activity	Analyses focusing on profiles or patterns of movement.
Genetic statistics	
Population‐level genetic structure	Genetic differentiation metrics using population allele frequencies.
Individual‐level spatial genetic structure	Spatial analyses taking individuals as the focal unit.
Genetic tagging	Mark–recapture approach using the genetic identity of each individual.
Individual assignment to populations	Process of genetically assigning individuals to putative populations of origin, according to genetic similarities between individuals.
Genetic covariates of dispersal data	Association analyses between individual genetic diversity and observed dispersal behavior.
Taxonomic classification	Use of genetic material for taxonomic classification.
Gene expression	Analysis of mRNA gene expression patterns.
Pathogen screen	Screen of the genetic material to detect pathogen genes.

*Note* : Description of the categories within each variable included in the data analysis. The categories for the research aim were extracted from the Shafer et al. ( 2016 ) paper, except for the aim called “mating systems as drivers of movement.”
